# Different stages of chronic kidney disease are associated with physical performance in adults over 60 years

**DOI:** 10.3389/fpubh.2022.963913

**Published:** 2022-09-09

**Authors:** Peiyu Song, Xinghong Xu, Yinjiao Zhao, Minghong Gu, Xiaoyu Chen, Hui Zhang, Xinze Wu, Chen Yu, Jianying Niu, Wei Ding, Suhua Zhang, Qi Guo

**Affiliations:** ^1^Jiangwan Hospital of Shanghai Hongkou District, Shanghai University of Medicine and Health Science Affiliated First Rehabilitation Hospital, Shanghai, China; ^2^Shanghai Yongci Rehabilitation Hospital, Shanghai, China; ^3^Department of Rehabilitation Medicine, Shanghai University of Medicine and Health Sciences, Shanghai, China; ^4^Department of Nephrology, Tongji Hospital, School of Medicine, Tongji University, Shanghai, China; ^5^Department of Nephrology, The Fifth People's Hospital of Shanghai, Fudan University, Shanghai, China; ^6^Department of Nephrology, Shanghai Ninth People's Hospital, Shanghai Jiao Tong University School of Medicine, Shanghai, China; ^7^Suzhou Kowloon Hospital, Shanghai Jiao Tong University School of Medicine, Suzhou, China

**Keywords:** sarcopenia, chronic kidney disease, muscle mass, muscle function, older adults

## Abstract

**Objective:**

The purpose of this study was to determine the association between different stages of chronic kidney disease (CKD) and sarcopenia and its components in the Chinese older population.

**Methods:**

The study comprised of 2,213 participants aged ≥ 60 years (1,025 men; mean age: 70.7 years) recruited from Shanghai who were invited to participate in a comprehensive geriatric assessment. Sarcopenia was defined according to the AWGS 2019 consensus update on sarcopenia diagnosis criteria. The glomerular filtration rate (GFR) was estimated using the equation that originated from the CKD-EPI equation, the stages of CKD are classified according to the Kidney Disease—Improving Global Outcomes (KDIGO).

**Results:**

The overall prevalence of sarcopenia was 19.0%, which increased with the severity of CKD. The prevalence of sarcopenia in patients with CKD 3–4 and kidney failure was significantly higher than that in normal and CKD 1–2 (*p* < 0.05). In logistic regression analysis model, compared with normal and CKD 1 patients, kidney failure was significantly associated with the increased risk of sarcopenia and low grip strength (*p* < 0.05); CKD 2, CKD 3–4 and kidney failure groups were significantly associated with an increased risk of low walking speed (*p* < 0.05), respectively; while the association between CKD and muscle mass was not shown.

**Conclusions:**

In our study, only decreased physical performance, as represented by walking speed, was significantly associated with increased CKD severity. This may improve the evidence for the prevention and intervention of sarcopenia in patients with CKD.

## Introduction

Sarcopenia is an age-related loss of muscle mass, strength and physical performance that reduces mobility and quality of life, is significantly associated with a poor prognosis of disability and death, and can lead to fall-related injuries requiring costly hospitalization and long-term rehabilitation (1, 2). Many epidemiological studies have shown that the prevalence of sarcopenia in the Asian older adult population ranges from 7.3 to 12.0% (3). Aging is a serious challenge in China, where the population aged 60 or older reached 230 million in 2016. This number continues to increase and is expected to reach approximately 418 million by 2035 (4). With the rapid rise of the elderly population, the number of patients with sarcopenia will increase greatly, which will bring a great burden to the society and economy.

Chronic kidney disease (CKD) is a catabolic state known to be associated with protein depletion and multiple metabolic disorders, and muscle synthesis may also be reduced (5). Epidemiological studies have shown that muscle wasting is prevalent in patients with CKD, that muscle mass loss is more severe and occurs earlier than in the same age group, and that these changes in body composition may be associated with long-term prognosis in this population (6, 7). Although some studies have shown that there is an association between CKD and sarcopenia, and sarcopenia is more common in patients with kidney failure (8). However, due to sample size and other reasons, the association with sarcopenia according to different stages of kidney disease patients was not included in the study, especially the lack of CKD stage 5 and dialysis patients (9, 10). The diagnosis of sarcopenia includes the reduction of skeletal muscle mass, strength and physical performance, and muscle function as an important component of sarcopenia has been included as a diagnostic prerequisite in the European consensus on sarcopenia, however, most of the current studies have gathered on the association between muscle mass and CKD, and the association between the components of sarcopenia in different stages of CKD has not been further shown in studies (9, 11).

Considering the increasing prevalence of CKD, the aim of this study was to determine the association between different stages of chronic kidney disease and sarcopenia in the Chinese older population. In addition, associations between the stages of CKD and sarcopenia and its components were compared.

## Methods

### Study participants

This cross-sectional study recruited older adults ≥60 years of age living in Shanghai, China who had joined China's national free physical examination program between 2019 and 2021. This study included two population cohorts, the community-based elderly population of the Adult Physical Fitness and Health Cohort Study (APFHCS) (ChiCTR1900024880) and kidney failure population of seven hemodialysis units (ChiCTR1900027039). Participants with the following conditions were excluded from the study: (1) unable to communicate with interviewers or to grant informed consent, (2) no blood samples were collected, (3) major neurological disorders including dementia, malignancies, (4) serious physical illness (joint replacement, amputation) that hampered their completion of the functional fitness tests. Following these exclusions, the final analyzed population comprised 2,213 subjects (men 1,025). The older adults of normal and CKD1-4 groups (*n* = 1844) were from the community, and patients with kidney failure (*n* = 369) were from seven hemodialysis centers and had undergone stable hemodialysis for at least 3 months. The study was approved by the Ethics Committee of Shanghai University of Medicine and Health Sciences and the methods were carried out in accordance with the principles of the Declaration of Helsinki. All participants were informed and signed consent prior to enrollment in the study.

### Baseline variable

All the participants were invited to a face-to-face interview to answer a standardized questionnaire after they completed their medical examination and performance-based assessments (12). The questionnaire regarding their lifestyle and disease history included questions about age, sex, occupation, educational level, marital status, family income, smoking habits (current smoker or not), drinking habits (drinking alcohol once a week, drinking in the past, and never drinking were all considered as no drinking) and physical activity level. Anthropometric parameters (including height and weight) were measured by trained personnel using standardized protocols. Physical activity was assessed using the short form of the International Physical Activity Questionnaire (IPAQ) (13). Medical history was also recorded, including diabetes, hypertension, stroke, dyslipidemia, and coronary heart disease (CHD).

### Assessment of sarcopenia

Sarcopenia was defined according to the AWGS 2019 consensus update on sarcopenia diagnosis criteria (3), in which a person who has low muscle mass, low muscle strength, and/or low physical performance was identified as having sarcopenia. Low muscle mass was defined as relative skeletal muscle mass index [appendicular skeletal muscle mass (ASM)/height (Ht)^2^] < 7.0 kg/m^2^ and 5.7 kg/m^2^ for men and women, respectively. Low muscle strength was defined as grip strength <28 kg or <18 kg in men and women, respectively. Low physical performance was defined as walking speed <1.0 m/s for both men and women.

Muscle mass was measured using a direct segmental multi-frequency bioelectrical impedance analysis (BIA) (In-Body 770; Biospace Co., Ltd, Seoul, Korea). Muscle strength was quantified using a handheld dynamometer (GRIP-D; Takei Ltd, Niigata, Japan). Grip strength is measured by the standard method of dominant hand in the elderly population, and it is measured by the side without fistula in patients with kidney failure. Participants were asked to exert their maximum effort twice and the strongest grip strength was recorded. To measure walking speed, two photocells connected to a recording chronometer were placed at the beginning and the end of a 4-meter course at the site clinic. Participants were instructed to stand with both feet touching the starting line and to begin walking at their usual pace after a verbal command was given. The time between activation of the first and the second photocell was measured and the average speed of two walks was recorded.

### Analysis of blood samples

A blood sample was obtained from the antecubital vein from patients who fasted overnight for at least 10 h. Blood sample analysis and blood pressure collection methods have been explained in our previous studies (14).

### Glomerular filtration rate assessment

The glomerular filtration rate (GFR) was estimated using the equation that originated from the CKD-EPI equation, which is more accurate than the equation from the MDRD Study group equation (15).

### CKD stage

According to the Kidney Disease—Improving Global Outcomes (KDIGO) classification, patients with eGFR ≥ 90 mL/min/1.73 m^2^ were classified as normal and CKD 1, eGFR between 60 and 89 mL/min/1.73 m^2^ as CKD 2, eGFR between 15 and 59 mL/min/1.73 m^2^ as CKD 3–4, and eGFR <15 mL/min/1.73 m^2^ as CKD 5, kidney failure (16).

### Statistical analyses

Characteristics of individuals at baseline were reported as medians (25–75th) percentiles for IPAQ; other continuous variables were presented as the means and standard deviation (SD), and categorical variables were expressed as percentages. We compared baseline patients' characteristics according to eGFR categories by Chi-Squared tests for categorical variables and one-way analysis of variance for continuous variables. Logistic regression analyses were used to assess the association between sarcopenia along with its individual components and CKD. Model 1 was adjusted for age and sex. Model 2 was adjusted for model 1 covariates plus BMI, widowed, farming, illiteracy, smoking, drinking, IPAQ, diabetes, hypertension, dyslipidemia, stroke and CHD. All statistical analyses were performed using SPSS v 26.0 (SPSS Inc, Chicago, IL), and *P*-values of < 0.05 were considered statistically significant.

## Results

Baseline characteristics of subjects were presented in Table 1 (1,025 men, mean age: 70.7 ± 6.5 years). Patients were classified into four groups according to CKD stages, and the number of patients in each group was 235 (10.6%), 1,380 (62.4%), 229 (10.3%), and 369 (16.7%), respectively. In the overall population, age, gender, BMI, education level, occupation, eGFR, smoking, drinking, grip strength, walking speed, body fat mass, body fat ratio, hypertension, diabetes, dyslipidemia, stroke and physical activity level were significantly different between the four groups (*p* < 0.05). The age and muscle mass of CKD 3–4 group and kidney failure subjects were significantly larger than those of normal and CKD 1 group subjects, while the grip strength was the opposite. Current drinkers and farmers' occupation were significantly lower in kidney failure than in the other three groups, and the prevalence of hypertension, diabetes and stroke was significantly higher than in the other three groups (*p*< 0.05).

**Table 1:** Baseline data classified by CKD stage.

	**Normal and CKD 1**	**CKD 2**	**CKD 3-4**	**Kidney failure**	***P*** **-value**
	**(*n* = 235)**	**(*n* = 1380)**	**(*n* = 229)**	**(*n* = 369)**	
Age (years)	68.1 ± 4.7	70.3 ± 6.6	72.4 ± 7.1_a_	72.4 ± 5.8_a_	<0.001
**Gender** ***, n(%)***					<0.001
Male	23(9.8)	759(55.0)	29(12.7)	214(58.0)	
Female	212(90.2)	621(45.0)	200(87.3)	155(42.0)	
BMI(kg/m^2^)	24.0 ± 3.6	24.4 ± 3.5	25.3 ± 4.0_a_	23.4 ± 3.6_a__b_	<0.001
Widowed (%)	51(21.7)	253(18.3)	43(18.8)	55(14.9)	0.195
Illiteracy*, n(%)*	39(16.6)	347(25.1)	78(34.1)	33(8.9)_a__b__c_	<0.001
Farmer*, n(%)*	157(66.8)	923(66.9)	167(72.9)	42(11.4)_a__b__c_	<0.001
eGFR(ml/min per 1.73 m^2^)	94.4 ± 4.0	76.3 ± 8.2	53.2 ± 8.4_a_	4.4 ± 1.5ab	<0.001
Smoking*, n(%)*	8(3.4)	361(26.2)	59(25.8)_a_	54(14.6)	<0.001
Drinking*, n(%)*	35(14.9)	409(29.6)_a_	75(32.8)_a_	30(8.1)_a__b__c_	<0.001
SMI (kg/m^2^)	6.32 ± 0.91	7.00 ± 1.10	6.49 ± 1.12_a_	6.64 ± 1.15_a_	0.260
WS (m/s)	1.09 ± 0.18	1.04 ± 0.23	0.95 ± 0.21_a_	0.85 ± 0.31_a__b_	<0.001
Grip (kg)	22.0 ± 7.3	26.7 ± 9.1	20.7 ± 6.4_a_	21.2 ± 7.2_a_	<0.001
Body fat mass (kg)	18.3 ± 6.5	18.9 ± 7.3	22.4 ± 8.1_a__b_	20.6 ± 7.4_a__b__c_	<0.001
Body fat percentage(%)	30.8 ± 7.5	28.9 ± 8.5_a_	35.1 ± 8.0_a__b_	32.4 ± 8.6_a__b__c_	<0.001
**Diseases** ***, n(%)***					
diabetes	46(19.6)	238(17.2)	51(22.3)	138(37.4)_a__b__c_	<0.001
hypertension	143(60.9)	795(57.6)	149(65.1)_b_	342(92.7)_a__b__c_	<0.001
dyslipidemia	44(18.7)	283(20.5)	65(28.4)_a__b_	133(36.0)_a__b_	<0.001
stroke	54(23.0)	244(17.7)	35(15.3)	122(33.1)	<.0.001
CHD	82(34.9)	409(29.6)	67(29.3)	120(32.5)	0.607
IPAQ(Met/wk)	5502(2688–10986)	3360(1386–7143)	2543(734–5779)	1245(347–2772)_a__b_	<0.001

BMI, body mass index; SMI, skeletal muscle mass index; WS, 4 meters walking speed; IPAQ, International Physical Activity Questionnaire; eGFR, estimated glomerular filtration rate; Data are presented as mean ± SD or n (%).

a Compared with normal and CKD 1 groups, P < 0.05;

b Compared with CKD 2 groups, P < 0.05;

c Compared with CKD 3–4 groups, P < 0.05.

**Figure 1:**
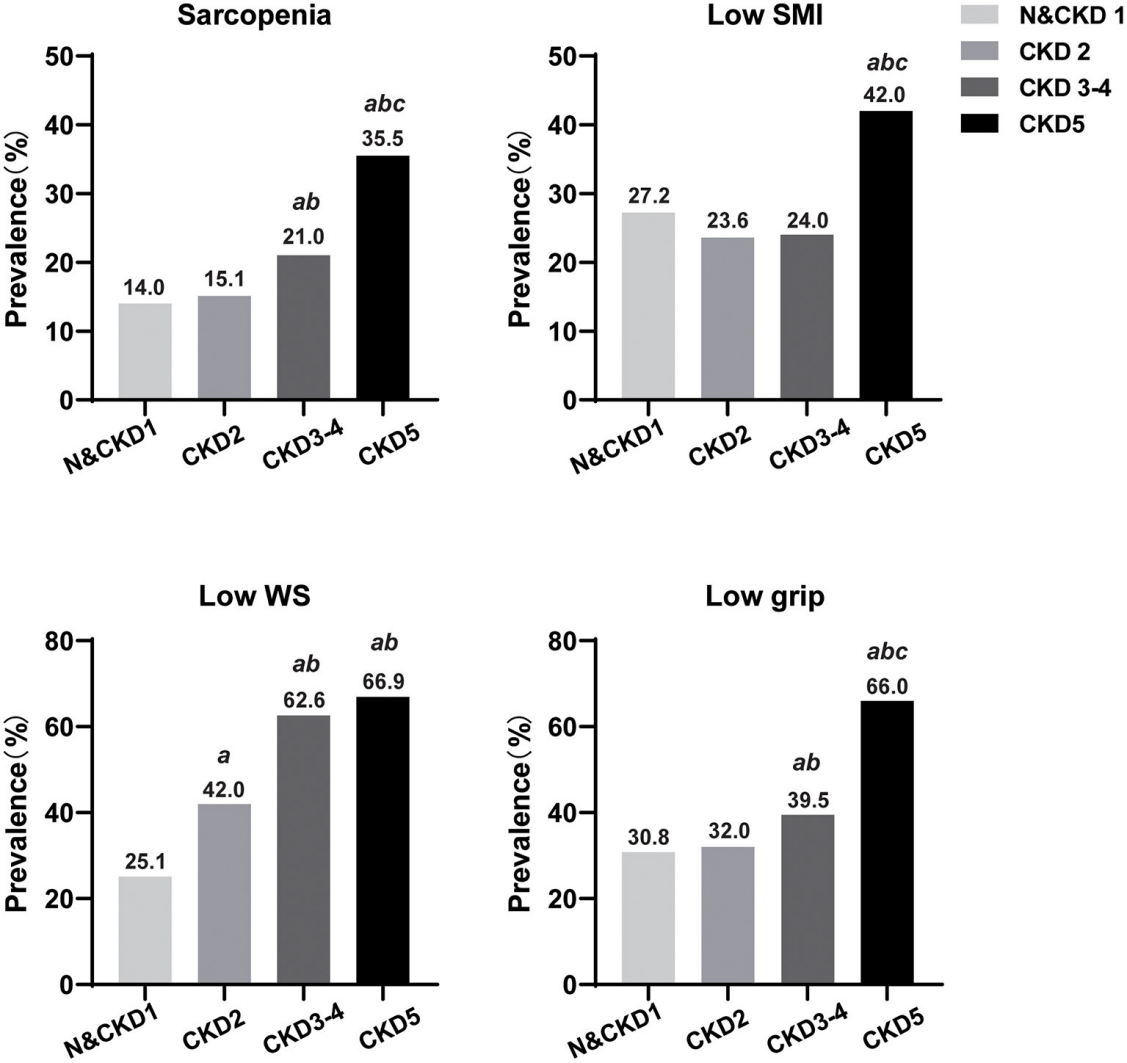
Prevalence of sarcopenia and its components according to chronic kidney disease stage. N, normal; CKD, chronic kidney disease; SMI, skeletal muscle mass index; WS, walking speed. a Compared with normal and CKD 1 groups, *P* < 0.05; b Compared with CKD 2 groups, *P* < 0.05; c Compared with CKD 3–4 groups, *P* < 0.05.

The prevalence of sarcopenia and its components classified by CKD stages was shown in Figure 1. The prevalence of sarcopenia, low walking speed and low grip strength generally increased with the severity of CKD, with the prevalence of sarcopenia significantly higher in patients with CKD 3–4 group than in normal and CKD 1–2 group patients, and the prevalence of sarcopenia significantly higher in kidney failure patients than in the other three groups. The prevalence of low SMI in kidney failure group were significantly higher than that in normal and CKD 1–4 group patients; the prevalence of low walking speed in CKD 3–4 and kidney failure groups was significantly higher than that in normal and CKD 1–2 group patients; patients with CKD 3–4 had a higher prevalence of low grip strength than normal and CKD 1–2, and patients with kidney failure had a significantly higher prevalence of low grip strength than normal and CKD 1 group (*p* < 0.05).

**Figure 2:**
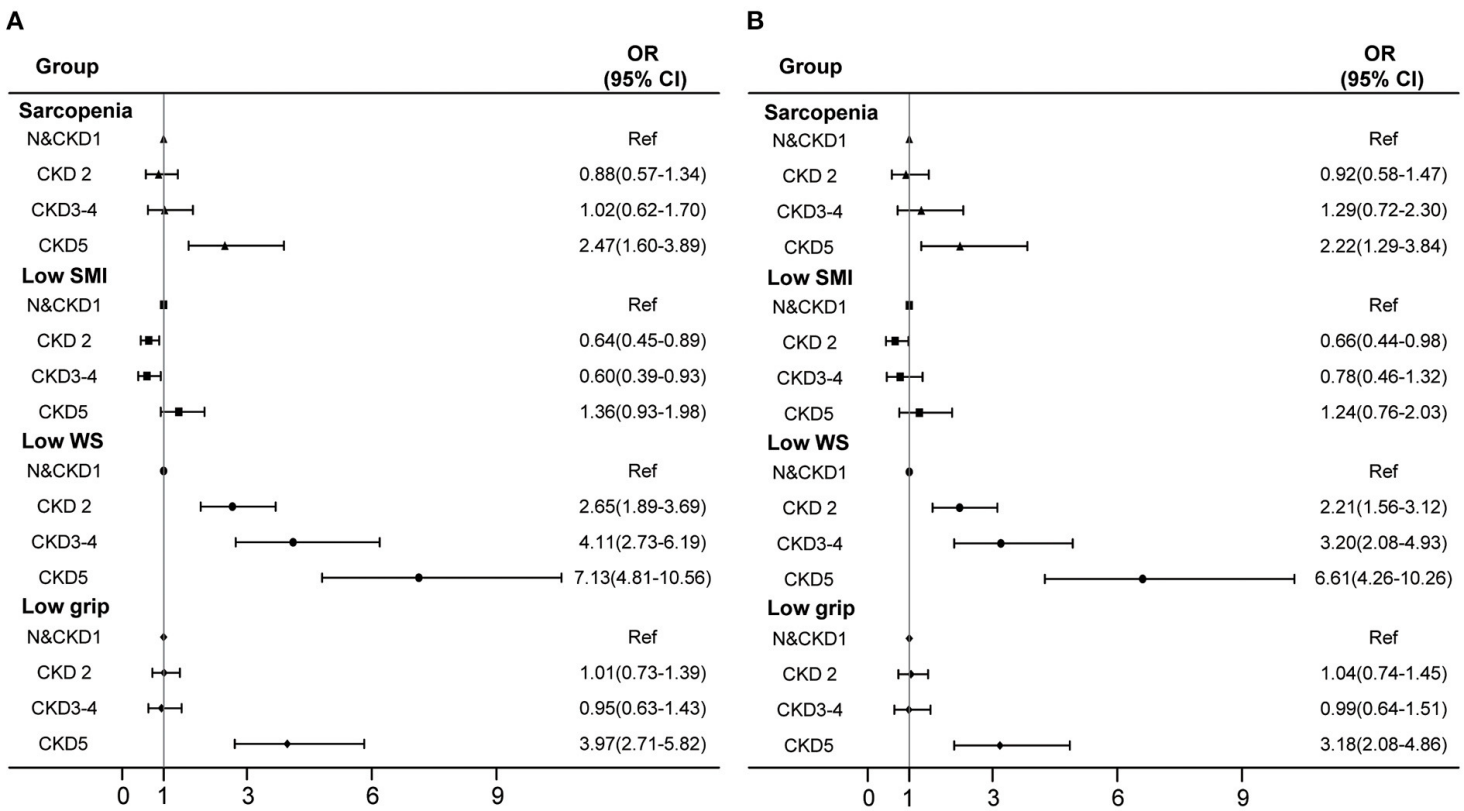
Association of different CKD stages with sarcopenia and its components. **(A)** crude model. **(B)** adjusted with age, sex, BMI, smoking, drinking, occupation, widowed, education, physical activity level, hypertension, diabetes, dyslipidemia, CHD and stroke. N, normal; CKD, chronic kidney disease; SMI, skeletal muscle mass index; WS, walking speed; CI, confidence interval; OR, odds ratio.

The association between different CKD stages and sarcopenia and its components is shown in Figure 2. In the adjusted logistic models, in patients with different CKD stages, compared with normal and CKD 1 group patients, kidney failure was significantly associated with the increased risk of sarcopenia and low grip strength. The odds ratio (OR) values (95% CI) were 2.22 (1.29–3.84) and 3.18 (2.08–4.86) respectively; compared with normal and CKD 1 patients, CKD 2, CKD 3–4 and kidney failure groups were significantly associated with an increased risk of low walking speed, with OR values (95% CI) of 2.21 (1.56–3.12), 3.20 (2.08–4.93) and 6.61 (4.26–10.26), respectively. There was no significant association between low muscle mass and different CKD stages.

## Discussion

This study examined the association of CKD of varying severity with sarcopenia and its components in a larger population. We found that the prevalence of sarcopenia increased with the severity of CKD, especially in patients with kidney failure. However, after multivariate adjustment, the risk of decreased physical performance represented by walking speed increased gradually with the increase of the severity of CKD. The risk of sarcopenia and low grip strength increased significantly only in kidney failure, and low muscle mass was not significantly associated with different stages of CKD.

Sarcopenia is a geriatric disease that has gained widespread attention for its significant impact on activity limitation, disability and even death in the older adults (3). In recent years, sarcopenia has gained attention in patients with CKD due to the accelerated protein catabolism of the disease process itself and low energy and protein intake in this population (17). However, in a study of sarcopenia in patients with CKD, there was no significant association between continuous eGFR and sarcopenia (18), this suggests that observing the correlation between CKD and sarcopenia may need to be observed in detail according to different stages of renal function. In the latest study to observe the association between sarcopenia and CKD at different stages, according to the grouping of eGFR, it was found that the risk of sarcopenia was significantly increased in male patients with CKD 3–5, and no significant association was found in female patients (11). Another study further characterized the staging of CKD and found that the risk of sarcopenia was significantly higher in patients with eGFR <45 mL/min/1.73 m^2^ (19). These studies suggest that the degree of association of sarcopenia may not be consistent across different stages of CKD, however, due to sample size and inclusion criteria, the number of patients with different stages of CKD in these studies was small, particularly in patients with CKD of higher severity. In our study, two population-based cohorts of older adults and CKD were combined and divided into four groups according to their renal function status. After multifactor adjustment, the risk of sarcopenia increased significantly only in patients with kidney failure, which may be related to the way that patients with kidney failure often need dialysis treatment (20), which provides further evidence for the prevention and treatment of sarcopenia in patients with chronic renal disease.

Patients with CKD exhibit a catabolic state, which is associated with protein depletion and multiple metabolic imbalances, hence the age-related decline in skeletal muscle mass confounded with the CKD disease state. Several studies have shown that muscle wasting in CKD is progressive and frequently observed (21). However, studies on CKD and low muscle mass found that low muscle mass was not associated with the risk of CKD in men, but with the reduced risk of CKD in women, and had no correlation with the onset and progression of CKD (22). Another longitudinal study, which lasted for 16 years, showed that there was no significant difference in the risk of muscle atrophy by simple CKD, and the risk of muscle atrophy increased significantly after combined diabetes (32). Therefore, the association between muscle mass and CKD may be inconsistent in different gender and complication status. In our study, there was no significant association between different stages of chronic kidney disease and low muscle mass. This may be because when our elderly patients with CKD and kidney failure were recruited, the selected patients had no obvious symptoms and were in good functional condition, which may have missed the coverage of the population. Secondly, we use bioelectrical impedance method to evaluate muscle mass. There is still no algorithm for accurate whole-body estimation in patients with obesity or changes in body fluid state (these two cases exist in CKD), which may also be an important reason why there is no significant difference in muscle mass in patients with CKD at different stages in our study. Future studies need to be extended to patients with symptomatic CKD to further reveal the association between different stages of CKD and muscle mass.

Sarcopenia is a common disease in the elderly, the most significant change is the decline of muscle mass and function (2). Muscle function includes muscle strength and physical performance (23). In a large study, the risk of low grip strength was significantly higher in patients with CKD, however, the association between grip strength and eGFR in the scatter plot analysis was not simply a linear positive correlation. Furthermore, only the mean grip strength and prevalence of low grip strength in patients with different stages of CKD were presented, and no statistical inferences were made about the association between low grip strength and different stages of CKD (33). In our study, after further subdividing the stages of CKD, the risk of low grip strength in patients with kidney failure is significantly increased, which may be due to the higher impact on muscle strength in more severe CKD, while other studies combined CKD after stage 3, in our study, the risk of low grip strength was also significantly increased in patients after CKD 3 (adjusted OR value 1.78, *P* < 0.001), thus the specific effects of different stages are not reflected. Secondly, in our study, patients with kidney failure were treated with hemodialysis, and some studies have shown that hemodialysis is an independent risk factor of muscle weakness (24). Therefore, in patients with high severity of CKD and hemodialysis, the risk of low muscle strength is significantly increased, which may be a further supplement to the current study.

Interestingly, the risk of poor physical performance represented by walking speed is gradually increasing in different stages of CKD. This is basically consistent with the association between different stages of CKD and physical performance observed in previous studies. Compared with normal and CKD 1–2, patients with CKD 3 and CKD 4–5 have an increased risk of low walking speed (OR values of 1.13 and 3.39, *p* < 0.05, respectively) (25). On the contrary, a cross-sectional study showed there were no significant association between low walking speed and CKD (26). The discrepancy may mainly be due to the age of participants. The prevalence of slow walking speed increases with age, and the average age of the above study (50.9 years) was lower than our study (70.7 years). The explanation for the association between CKD and low walking speed could be the direct impact of kidney function on walking speed. Even though in early-stage, impairments in kidney function were also associated with the accumulation of neurotoxins that have been shown to cause axonal loss with secondary or predominant demyelination, b2-microglobulin deposition in joints and connective tissue, and increased levels of inflammatory cytokines, which can all result in physical function loss (27, 28). Walking speed assessment has the important advantages of brevity, simplicity and reproducible (29). Therefore, we suggested walking speed test might be seen as a screening tool for early detection of functional limitations, providing an informative and potentially actionable functional status in the CKD population.

It is worth noting that in our study, the prevalence of CKD in different genders has different trends. In CKD3-4 stage, women are much higher than men. However, in the stage of kidney failure, the prevalence of men is higher than women. These data are consistent with the trend of the results of the current study. The incidence rate of women in CKD is higher than that of men, and lower than that of men in renal replacement therapy (30). The effect of prolonged life expectancy on the natural decline of GFR with age, and the improper use of GFR equation may lead to over diagnosis of CKD, which may be part of the reason for the increase in the prevalence of CKD in women. Among patients with kidney failure, elderly women seem to prefer conservative treatment rather than renal replacement therapy, which also reflects that men are higher than women in dialysis patients. However, it is worth considering that there is a large difference in the ratio between men and women in patients with CKD, which may be due to the estimation of GFR or the defect in our definition of normal GFR, resulting in the over diagnosis of female CKD. Studies have shown that even the current more accurate CKD-EPI equation has a large error in estimating the glomerular filtration rate of women (31). Therefore, more accurate estimation of GFR may have great clinical significance for correct classification in the future.

Our study has several strengths. We combined two independent population cohorts to supplement the samples of patients with different stages of CKD and comprehensively show the relationship between CKD and sarcopenia at each stage. Our results can supplement evidence on the relationship between physical performance and kidney function among older adults, which could contribute to providing appropriate treatment for the prevention of CKD. Although this study has its advantages, it still has some limitations. First, this is a cross-sectional study, so we cannot determine the causal relationship between CKD and sarcopenia. Secondly, our population are all participants with good functional status, who can participate in functional evaluation and have no obvious symptoms. Third, bioelectrical impedance method is used to evaluate muscle mass. Due to the large change of hydration state, this method may have errors in CKD patients. Future studies should expand the number of participants, add more relevant data and carry out more accurate and comprehensive assessments to further determine and enhance our understanding of the relationship between sarcopenia and CKD.

## Conclusion

In conclusion, we found that the prevalence of sarcopenia increased significantly with the development of CKD severity. With regard to its components, only the decrease in physical performance, represented by walking speed, was significantly associated with increased CKD severity, while the association with muscle mass is not shown. These findings inform clinical decision making and facilitate the development of therapies to prevent sarcopenia in older patients with CKD. Future studies should test the effectiveness of relevant interventions in older patients with CKD.

## Data availability statement

The raw data supporting the conclusions of this article will be made available by the authors, without undue reservation.

## Ethics statement

The studies involving human participants were reviewed and approved by Shanghai University of Medicine and Health Sciences. The patients/participants provided their written informed consent to participate in this study.

## Author contributions

QG, SZ, and PS conceived the concept and design of the study. XC, HZ, XW, and YZ collected and assembled the data. YZ, JN, WD, and HZ analyzed and interpreted the data. YZ and PS drafted the article or revising it critically for important intellectual content. SZ and QG provided administrative support. XX and MG participated in data processing, statistical analysis, and revision of the manuscript. All authors approved the final version.

## Funding

This work was supported by the National Natural Science Foundation of China (No. 82172552).

## Conflict of interest

The authors declare that the research was conducted in the absence of any commercial or financial relationships that could be construed as a potential conflict of interest.

## Publisher's note

All claims expressed in this article are solely those of the authors and do not necessarily represent those of their affiliated organizations, or those of the publisher, the editors and the reviewers. Any product that may be evaluated in this article, or claim that may be made by its manufacturer, is not guaranteed or endorsed by the publisher.

## References

[1] Chin SO Rhee SY Chon S Hwang YC Jeong IK Oh S. Sarcopenia is independently associated with cardiovascular disease in older Korean adults: the Korea National Health and Nutrition Examination Survey (KNHANES) from 2009. PLoS One. (2013) 8:e60119. 10.1371/journal.pone.0060119 23533671 PMC3606314

[2] Larsson L Degens H Li M Salviati L Lee YI Thompson W. Sarcopenia: aging-related loss of muscle mass and function. Physiol Rev. (2019) 99:427–511. 10.1152/physrev.00061.2017 30427277 PMC6442923

[3] Chen LK Woo J Assantachai P Auyeung TW Chou MY Iijima K. Asian Working Group for Sarcopenia: 2019 consensus update on sarcopenia diagnosis and treatment. J Am Med Dir Assoc. (2020) 21:300–7. e302. 10.1016/j.jamda.2019.12.012 32033882

[4] Li X Fan L Leng SX. The aging tsunami and senior healthcare development in China. J Am Geriatr Soc. (2018) 66:1462–8. 10.1111/jgs.15424 29974937

[5] Wang XH Mitch WE. Mechanisms of muscle wasting in chronic kidney disease. Nat Rev Nephrol. (2014) 10:504–16. 10.1038/nrneph.2014.112 24981816 PMC4269363

[6] Domanski M Ciechanowski K. Sarcopenia: a major challenge in elderly patients with end-stage renal disease. J Aging Res. (2012) 2012:754739. 10.1155/2012/754739 22536505 PMC3321443

[7] Sharma D Hawkins M Abramowitz MK. Association of sarcopenia with eGFR and misclassification of obesity in adults with CKD in the United States. Clin J Am Soc Nephrol. (2014) 9:2079–88. 10.2215/CJN.02140214 25392147 PMC4255396

[8] Souza VA Oliveira D Mansur HN Fernandes NM Bastos MG. Sarcopenia in chronic kidney disease. J Bras Nefrol. (2015) 37:98–105. 10.5935/0101-2800.20150014 25923756

[9] An JN Kim JK Lee HS Kim SG Kim HJ Song YR. Late stage 3 chronic kidney disease is an independent risk factor for sarcopenia, but not proteinuria. Sci Rep. (2021) 11:18472. 10.1038/s41598-021-97952-7 34531464 PMC8446068

[10] Yu MD Zhang HZ Zhang Y Yang SP Lin M Zhang YM. Relationship between chronic kidney disease and sarcopenia. Sci Rep. (2021) 11:20523. 10.1038/s41598-021-99592-3 34654871 PMC8520007

[11] Moon SJ Kim TH Yoon SY Chung JH Hwang HJ. Relationship between stage of chronic kidney disease and sarcopenia in Korean aged 40 years and older using the Korea National Health and Nutrition Examination Surveys (KNHANES IV-2, 3. and V-1, 2), 2008-2011. PLoS One. (2015) 10:e0130740. 10.1371/journal.pone.0130740 26083479 PMC4470593

[12] Song P Han P Zhao Y Zhang Y Wang L Tao Z. Muscle mass rather than muscle strength or physical performance is associated with metabolic syndrome in community-dwelling older Chinese adults. BMC Geriatr. (2021) 21:191. 10.1186/s12877-021-02143-8 33740914 PMC7980667

[13] Craig CL Marshall AL Sjostrom M Bauman AE Booth ML Ainsworth BE. International physical activity questionnaire: 12-country reliability and validity. Med Sci Sports Exerc. (2003) 35:1381–95. 10.1249/01.MSS.0000078924.61453.FB 12900694

[14] Han P Yu H Ma Y Kang L Fu L Jia L. The increased risk of sarcopenia in patients with cardiovascular risk factors in Suburb-Dwelling older Chinese using the AWGS definition. Sci Rep. (2017) 7:9592. 10.1038/s41598-017-08488-8 28851881 PMC5575090

[15] Levey AS Bosch JP Lewis JB Greene T Rogers N Roth D. A more accurate method to estimate glomerular filtration rate from serum creatinine: a new prediction equation. modification of diet in renal disease study group. Ann Intern Med. (1999) 130:461–70. 10.7326/0003-4819-130-6-199903160-00002 10075613

[16] Zhang L Wang F Wang L Wang W Liu B Liu J. Prevalence of chronic kidney disease in China: a cross-sectional survey. Lancet. (2012) 379:815–22. 10.1016/S0140-6736(12)60033-6 22386035

[17] Sabatino A Cuppari L Stenvinkel P Lindholm B Avesani CM. Sarcopenia in chronic kidney disease: what have we learned so far? J Nephrol. (2021) 34:1347–72. 10.1007/s40620-020-00840-y 32876940 PMC8357704

[18] Souza VA Oliveira D Barbosa SR Correa J Colugnati FAB Mansur HN. Sarcopenia in patients with chronic kidney disease not yet on dialysis: Analysis of the prevalence and associated factors. PLoS One. (2017) 12:e0176230. 10.1371/journal.pone.0176230 28448584 PMC5407780

[19] Chen X Han P Yu X Zhang Y Song P Liu Y. Relationships between sarcopenia, depressive symptoms, and mild cognitive impairment in Chinese community-dwelling older adults. J Affect Disord. (2021) 286:71–7. 10.1016/j.jad.2021.02.067 33714172

[20] Mori K. Maintenance of skeletal muscle to counteract sarcopenia in patients with advanced chronic kidney disease and especially those undergoing hemodialysis. Nutrients. (2021) 13. 10.3390/nu13051538 34063269 PMC8147474

[21] Workeneh BT Mitch WE. Review of muscle wasting associated with chronic kidney disease. Am J Clin Nutr. (2010) 91:1128S−32S. 10.3945/ajcn.2010.28608B 20181807

[22] Kruse NT Buzkova P Barzilay JI Valderrabano RJ Robbins JA Fink HA. Association of skeletal muscle mass, kidney disease and mortality in older men and women: the cardiovascular health study. Aging (Albany NY). (2020) 12:21023–36. 10.18632/aging.202135 33139582 PMC7695366

[23] Cruz-Jentoft AJ Sayer AA. Sarcopenia. Lancet. (2019) 393:2636–46. 10.1016/S0140-6736(19)31138-9 31171417

[24] Shirai N Yamamoto S Osawa Y Tsubaki A Morishita S Igarashi K. Comparison of muscle strength between hemodialysis patients and non-dialysis patients with chronic kidney disease. J Phys Ther Sci. (2021) 33:742–7. 10.1589/jpts.33.742 34658517 PMC8516613

[25] Wickstrom JF Sayles HR Graeff-Armas LA Yentes JM. The likelihood of self-reporting balance problems in those with advanced chronic kidney disease, slow gait speed, or low vitamin D. J Ren Nutr. (2019) 29:490–7. 10.1053/j.jrn.2018.10.011 30581062 PMC6586542

[26] Michishita R Matsuda T Kawakami S Tanaka S Kiyonaga A Tanaka H. The Association between unhealthy lifestyle behaviors and the prevalence of Chronic Kidney Disease (CKD) in middle-aged and older men. J Epidemiol. (2016) 26:378–85. 10.2188/jea.JE20150202 26947951 PMC4919483

[27] Galassi G Ferrari S Cobelli M Rizzuto N. Neuromuscular complications of kidney diseases. Nephrol Dial Transplant. (1998) 13 (Suppl. 7):41–7. 10.1093/ndt/13.suppl_7.41 9870436

[28] Yokoi H Yanagita M. Decrease of muscle volume in chronic kidney disease: the role of mitochondria in skeletal muscle. Kidney Int. (2014) 85:1258–60. 10.1038/ki.2013.539 24875545

[29] Cummings SR Studenski S Ferrucci L. A diagnosis of dismobility–giving mobility clinical visibility: a Mobility Working Group recommendation. JAMA. (2014) 311:2061–2. 10.1001/jama.2014.3033 24763978 PMC5012417

[30] Carrero JJ Hecking M Chesnaye NC Jager KJ. Sex and gender disparities in the epidemiology and outcomes of chronic kidney disease. Nat Rev Nephrol. (2018) 14:151–64. 10.1038/nrneph.2017.181 29355169

[31] Inker LA Levey AS Tighiouart H Shafi T Eckfeldt JH Johnson C. Performance of glomerular filtration rate estimating equations in a community-based sample of blacks and whites: the multiethnic study of atherosclerosis. Nephrol Dial Transplant. (2018) 33:417–25. 10.1093/ndt/gfx042 28505377 PMC6018782

[32] Lee C Kim HJ Chang TI Kang EW Joo YS Kim HW. Synergic association of diabetes mellitus and chronic kidney disease with muscle loss and cachexia: results of a 16-year longitudinal follow-up of a community-based prospective cohort study. Aging (Albany NY). (2021) 13:21941–61. 10.18632/aging.203539 34528898 PMC8507303

[33] Lee YL Jin H Lim JY Lee SY. Relationship Between Low Handgrip Strength and Chronic Kidney Disease: KNHANES 2014-2017. J Ren Nutr. (2021) 31:57–63. 10.1053/j.jrn.2020.03.002 32381354

